# Cost-effectiveness analysis of serplulimab combination therapy versus chemotherapy alone for patients with extensive-stage small cell lung cancer

**DOI:** 10.3389/fonc.2023.1259574

**Published:** 2024-01-11

**Authors:** Zhiwei Zheng, Hongcai Chen, Hongfu Cai

**Affiliations:** ^1^ Department of Pharmacy, Cancer Hospital of Shantou University Medical College, Shantou, China; ^2^ Department of Oncology Medicine, Cancer Hospital of Shantou University Medical College, Shantou, China; ^3^ Department of Pharmacy, Fujian Medical University Union Hospital, Fuzhou, China

**Keywords:** serplulimab, cost-effectiveness, extensive-stage, small cell lung cancer, chemotherapy

## Abstract

**Background:**

Serplulimab has shown promising results in the treatment of extensive-stage small cell lung cancer (ES-SCLC). This study aimed to evaluate the cost-effectiveness of serplulimab combination therapy compared to chemotherapy alone in patients with ES-SCLC from the Chinese healthcare system perspective.

**Methods:**

A partitioned survival model was developed to simulate the costs and outcomes of patients receiving serplulimab combination therapy or chemotherapy alone over a time horizon of 10 years. Data on overall survival, progression-free survival, and adverse events were obtained from the ASTRUM-005 randomized clinical trial. Costs were estimated from a healthcare system perspective and included drug acquisition, administration, monitoring, and management of adverse events. One-way and probabilistic sensitivity analyses were conducted to assess the impact of uncertainty on the results.

**Results:**

The base-case analysis showed that the combination of serplulimab and chemotherapy has demonstrated a significant increase in QALYs of 0.626 compared to chemotherapy alone. This improved outcome is accompanied by an additional cost of $10893.995. The ICER for incorporating serplulimab into the treatment regimen is $17402.548 per QALY gained. One-way sensitivity analysis confirmed the robustness of the findings. Probabilistic sensitivity analysis demonstrated that serplulimab combination therapy had a 97.40% high probability of being cost-effective compared to chemotherapy alone at the WTP thresholds.

**Conclusion:**

In contrast to chemotherapy as a standalone treatment, the addition of serplulimab to chemotherapy is believed to offer potential cost-effectiveness as a preferred initial therapeutic approach for patients with ES-SCLC in China.

## 1 Introduction

Small cell lung cancer (SCLC) is an extremely aggressive variant of lung cancer, accounting for approximately 15% of all cases (1). It is characterized by its notable rapid cell proliferation and ability to spread to distant sites (2). Unfortunately, the majority of individuals with SCLC are diagnosed at an advanced stage, resulting in a dismal 5-year survival rate of only 7% (3). Although the current primary treatment regimen consisting of platinum-based chemotherapy and etoposide is the standard first-line therapy, this therapeutic approach yields an average overall survival duration of approximately 10 months (4). The low survival rate in advanced cases highlights the urgent need for improved treatment strategies.

The introduction of immune checkpoint inhibitors (ICIs) has had a profound impact on the therapeutic landscape of extensive-stage small-cell lung cancer (ES-SCLC) (5). In recent years, the development of ICIs targeting programmed cell death protein 1 (PD-1) and programmed death ligand 1 (PD-L1) has significantly improved the efficacy of immunotherapy and enhanced patient survival (6). The incorporation of atezolizumab into the initial therapeutic approach for ES-SCLC has shown significant improvements in both overall survival (OS) and progression-free survival (PFS)when compared to exclusive administration of chemotherapy (7).

Furthermore, the combination of durvalumab and platinum-etoposide as a first-line treatment for ES-SCLC has demonstrated sustained improvement in overall survival compared to the standard treatment of platinum-etoposide alone (8). Additionally, the addition of adebrelimab to chemotherapy has shown considerable enhancement in overall survival rates without compromising the safety of patients diagnosed with ES-SCLC (9). These findings indicate the potential of immunotherapy agents in improving treatment outcomes for ES-SCLC patients. Moreover, a separate phase 3 trial investigated the effectiveness of the PD-1 inhibitor pembrolizumab when administered concurrently with chemotherapy and showed a notable extension in progression-free survival (10). These results support the potential of PD-L1 and PD-1 inhibitors in treating ES-SCLC, particularly when used alongside chemotherapy. However, it must be acknowledged that the clinical benefit observed with these ICIs is currently limited. Therefore, further research and development of new drugs are urgently needed to design more effective therapeutic strategies to address this aggressive disease.

A recently phase III trial (ASTRUM-005) examined the efficacy and safety of serplulimab when combined with chemotherapy in patients with previously untreated ES-SCLC.The findings of this trial demonstrated that the addition of serplulimab to chemotherapy led to a significant prolongation of progression-free survival (PFS) (with a median PFS of 5.8 months compared to 4.3 months in the chemotherapy-only group, hazard ratio [HR] = 0.47, 95% confidence interval [CI] 0.38-0.59). Furthermore, the combination therapy also resulted in a significant improvement in OS (with a median OS of 15.4 months compared to 10.9 months in the chemotherapy-only group, HR = 0.63, 95% CI 0.49-0.82) (11). The promising results obtained from this trial led to the granting of orphan drug status for serplulimab in the treatment of ES-SCLC by the US Food and Drug Administration (FDA). Additionally, serplulimab has also received approval for use as a first-line therapy for ES-SCLC in China.

While the clinical endpoints of the trial were met, it is crucial to consider the implications of cost-effectiveness when combining serplulimab with chemotherapy, as this approach incurs additional expenses compared to using chemotherapy alone. Hence, the primary aim of this study was to evaluate the cost-effectiveness of adding serplulimab to chemotherapy compared to chemotherapy alone as the first line treatment for patients with ES-SCLC from the Chinese healthcare system perspective. The health system perspective of pharmaceutical economics assessment holds significant advantages as it places primary emphasis on direct health care costs incurred within the healthcare system. By focusing on these costs, it avoids the challenges associated with collecting and evaluating the more elusive indirect costs. Furthermore, the health system perspective provides valuable insights into the efficiency of resource allocation within the broader healthcare system. This enables health system managers to effectively prioritize the promotion of medicines based on the economic assessment conducted at their specific level of authority.

## 2 Method

### 2.1 Model structure

**Figure 1:**
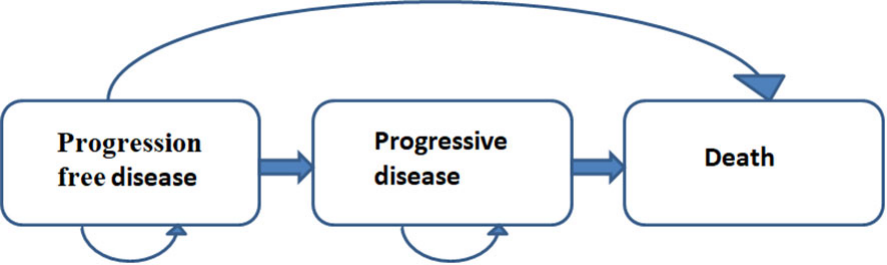
Partitioned survival model structure.

We have developed a partitioned survival model that utilizes a mutually exclusive trichotomous health state classification: progressive-free survival, progressive disease (PD), and death (**Figure 1**). The simulation model cycle of our model aligns with that of the ASTRUM-005 clinical trial, spanning three weeks. Moreover, the time horizon for our study was set at ten years, as previous research has shown a dismal five-year overall survival rate of only approximately 7% for extensive-stage small cell lung cancer.

To establish a threshold for willingness-to-pay (WTP), we adopted a value of $37,304.346 per quality-adjusted life year (QALY), which is equivalent to three times the national gross domestic product (GDP) in the year 2022 (12). This threshold serves as a benchmark for assessing the cost-effectiveness of interventions.

To conduct our modeling process, we utilized the TreeAge Pro 2011 software. This software offers robust capabilities for constructing and analyzing decision trees, which are central to our partitioned survival model.

### 2.2 Population and treatment

The study population included patients who met the inclusion criteria of the phase III ASTRUM-005 clinical trial. The recruitment period spanned from September 12, 2019, to April 27, 2021. The study included a total of 585 patients who were randomly assigned. The patients had an average age of 61.1 years (standard deviation, 8.67 years), and 104 of them (17.8%) were women. In the serplulimab group, 67.4% of the population was from Asia, 81.5% were men, and the average age was 63 years, while in the chemotherapy group, 70.9% of the population was from Asia, 83.7% were men, and the average age was 62 years. The baseline characteristics were found to be evenly distributed and comparable between the two study groups. Serplulimab was administered intravenously every 3 weeks at a dose of 4.5 mg/kg on day 1 of each treatment cycle. The placebo used in this study was appropriately matched to serplulimab. Additionally, all patients received etoposide at a dose of 100 mg/m^2^ on days 1, 2, and 3, as well as carboplatin at a dose determined by the area under the serum drug concentration-time curve of 5 mg/mL/min on day 1 of each treatment cycle, with a maximum dose of 750 mg. These medications were administered through intravenous infusions for a maximum of 4 cycles. Following the initial treatment phase, patients entered a maintenance therapy phase where they continued to receive serplulimab or placebo according to the treatment cycle.

In the ASTRUM-005 clinical trial, the results indicate that the group receiving serplulimab treatment had a median treatment duration of 5.6 months (range: 4.2 to 6.8 months), while the placebo group had a median treatment duration of 3.2 months (range: 2.9 to 4.2 months). After the initial occurrence of disease progression, a total of 172 patients (44.2%) in the serplulimab group and 85 patients (43.4%) in the placebo group received additional treatment. This subsequent treatment primarily consisted of irinotecan, another chemotherapy agent.

The optimal choice for third-line therapy following the failure of second-line therapy is currently uncertain. Therefore, in our study, supportive treatment was considered the most effective option when the disease progressed once again. Our survival model was developed considering grade 3 or 4 adverse events (AEs) that occurred at a rate exceeding 5% in both the serplulimab and chemotherapy treatment groups.

### 2.3 Survival transition probabilities

Survival data for each treatment group were extracted from the ASTRUM-005 trial using the GetData Graph Digitizer software. These extracted curves were then simulated in the R software using various distribution options, including weibull, log-logistic, log-normal, exponential, gompertz, and gamma distributions (13). The purpose of this analysis was to identify the most appropriate distribution for accurately simulating the survival curves. To determine the most appropriate distribution for simulating the survival curves, visual inspection and data analysis were conducted (14). Notably, based on the lowest values of the Akaike information criterion and Bayesian information criterion, the log-logistic distribution exhibited the highest level of fit to the clinical trial data. Consequently, the log-logistic distribution was selected as the optimal distribution for predicting the long-term survival status of patients in this study. **Supplementary Table 1** and **Supplementary Figure 1** provide additional details regarding the data and visual inspection conducted during the analysis process.

### 2.4 Cost and utility

This study aimed to conduct a comprehensive cost analysis to evaluate the direct medical expenses associated with cancer treatment. Specifically, the focus was on assessing the costs related to various aspects of cancer treatment, including drug cost, management of severe adverse events graded as 3 and 4, follow-up cost, subsequent treatment cost, and best supportive care cost. In order to determine the costs of medications, data from the China Health Industry Data Platform (https://data.yaozh.com/) were utilized, using the national median price as the reference point (15). Additionally, costs from other aspects of treatment were obtained from recent literature publications.

Costs were converted from Chinese Renminbi (RMB) to United States dollars (US$) using the average exchange rate of 6.73 RMB to 1 US$ in the year 2022 (16). To account for the time value of money, future costs and utilities were discounted at a rate of 5% (17). Chemotherapy doses were determined based on a standardized model that assumes a body weight of 60 kg, creatinine clearance of 70μmol/L and a body surface area of 1.72 m^2^.

The assessment of health-related quality of life is a crucial aspect in evaluating the impact of health conditions on individuals. Utility values are commonly employed to measure health-related quality of life, representing scores ranging from 0 to 1 that signify the poorest and healthiest states respectively. However, in the ASTRUM-005 clinical trial, there was a lack of utility data specifically related to healthy living. Therefore, it was necessary to rely on utility values reported in published literature. It is important to note that these selected utility values will also undergo rigorous sensitivity analysis to thoroughly examine their influence on the study’s findings.

Moreover, this study also takes into consideration the adverse effects of drug-related events on health-related quality of life.Both the associated costs and utility values are included in **Table 1**.

**Table 1:** Model parameters input data.

Parameters	Baseline value	Range		Distribution	Source
		Minimum	Maximum		
Log-logistic OS survival model					
Serplulimab group	γ=1.937;λ=0.00363	–	–	–	(11)
Chemotherapy group	γ=1.924;λ=0.00645	–	–	–	(11)
Log-logistic PFS survival model					
Serplulimab group	γ=2.100;λ=0.0129	–	–	–	(11)
Chemotherapy group	γ=2.520;λ=0.0152	–	–	–	(11)
Drug costs ($)					
Serplulimab (100mg)	812.209	609.382	1015.261	Gamma	(15)
Etoposide(100mg)	5.661	4.246	7.076	Gamma	(15)
Carboplatin(100mg)	24.215	18.161	30.269	Gamma	(15)
Treatment-emergent adverse event(Serplulimab group)					
Anemia	0.054	–	–	Gamma	(11)
Decreased white blood cell count	0.085	–	–	Gamma	(11)
Decreased neutrophil count	0.141	–	–	Gamma	(11)
Decreased platelet count	0.062				
Treatment-emergent adverse event(Chemotherapy group)					
Anemia	0.056	–	–	Gamma	(11)
Decreased white blood cell count	0.087	–	–	Gamma	(11)
Decreased neutrophil count	0.138	–	–	Gamma	(11)
Decreased platelet count	0.082	–	–	Gamma	
Cost of treatment-emergent adverse event per cycle					
Anemia	531.723	398.792	664.653	Gamma	(18)
Decreased white blood cell count	461.253	345.939	576.566	Gamma	(18)
Decreased neutrophil count	84.210	63.157	105.262	Gamma	(19)
Decreased platelet count	1054.000	790.500	1317.500	Gamma	(19)
Utility					
Anemia	0.073	0.055	0.091	Beta	(18)
Decreased white blood cell count	0.200	0.150	0.250	Beta	(18)
Decreased neutrophil count	0.200	0.150	0.250	Beta	(19)
Decreased platelet count	0.190	0.143	0.238	Beta	(19)
Progression-free disease	0.673	0.505	0.841	Beta	(20)
Progressive disease	0.473	0.355	0.591	Beta	(20)
Other parameters					
Subsequent therapy cost per cycle	854.050	640.538	1067.563	Gamma	(21)
Follow-up cost per cycle	55.600	41.700	69.500	Gamma	(22)
Best supportive care	359.524	269.643	449.405	Gamma	(22)
Body surface area(m^2^)	1.720	1.290	2.150	Gamma	(22)

### 2.5 Sensitivity analysis

To examine the potential factors influencing the incremental cost-effectiveness ratio (ICER), a comprehensive sensitivity analysis was conducted. Specifically, a one-way sensitivity analysis was performed by adjusting each input parameter by ±25% to assess its impact on the ICER. The outcomes of this analysis were visually represented using a tornado plot, which effectively showcased the magnitude of influence that each parameter exerted on the ICER.

Furthermore, to ensure the robustness of our findings, a probabilistic sensitivity analysis (PSA)was executed. This involved conducting 1000 Monte Carlo simulations, which allowed for a more comprehensive assessment of the uncertainties associated with the ICER. In this PSA, the cost factor was modeled utilizing a gamma distribution, while the utility value factor was captured using a beta distribution. To better present these results, scatter plots were employed, enabling a clear and concise depiction of the outcomes.

Collectively, these analyses offer valuable insights into the potential variability and uncertainties surrounding the resulting ICERs. By systematically assessing the influence of various parameters and conducting a probabilistic assessment, we are able to enhance the robustness and reliability of our findings, thereby providing a more comprehensive understanding of the effectiveness and cost implications of the intervention under investigation.

## 3 Result

### 3.1 The result of base case analysis

The addition of serplulimab to the treatment regimen for ES-SCLC has been found to have a significant positive impact on the QALYs, with a gain of 0.626 compared to chemotherapy alone. However, this improved outcome is accompanied by an additional cost of $10,893.995. Consequently, the ICER for incorporating serplulimab into the treatment regimen for ES-SCLC has been calculated to be $17,402.548 per QALY gained.

The ICER is an important measure used to assess the value for money of a healthcare intervention. In this case, the ICER of $17402.548 per QALY gained falls below the WTP threshold of $37,304.346 per QALY, suggesting that the combination therapy of serplulimab and chemotherapy may be considered a cost-effective option for managing ES-SCLC. **Table 2** presents the detailed findings from the base case investigation, providing a comprehensive overview of the results obtained.

**Table 2:** The outcomes of the base case analysis.

Parameters	Cost ($)	QALYs	Incremental cost ($)	Incremental QALY	ICER ($/QALY)
Serplulimab group	31020.152	1.172	10893.995	0.626	17402.548
Chemotherapy group	20126.157	0.546	NA	NA	NA

ICER, Incremental cost–effectiveness ratio; QALY, Quality-adjusted life year; NA, Not applicable.

### 3.2 Sensitivity analysis result

**Figure 2:**
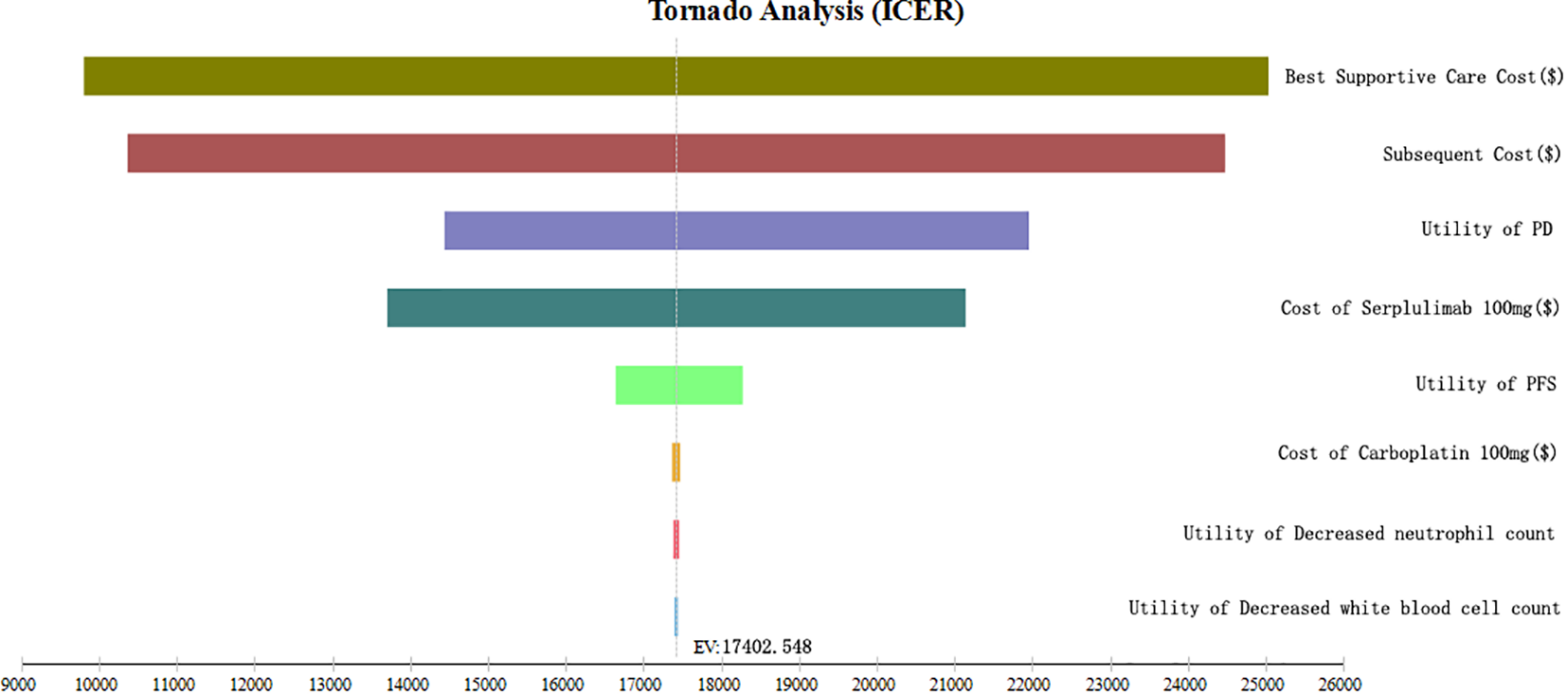
Tornado plot of one-way sensitivity analysis.

The tornado plot in **Figure 2** provides insights into the results of a one-way sensitivity analysis. It is noteworthy that the cost of best supportive care has the most significant impact on the ICER. Furthermore, several other factors, including the subsequent costs, the utility of PD, cost of serplulimab, and the PFS utility, also demonstrate some influence on the ICER. However, it is important to emphasize that varying these parameters within a ±25% range did not substantially alter the overall findings of the analysis. This observation is crucial as it indicates the robustness of the model and suggests that the results are not highly sensitive to changes in these parameters.

**Figure 3:**
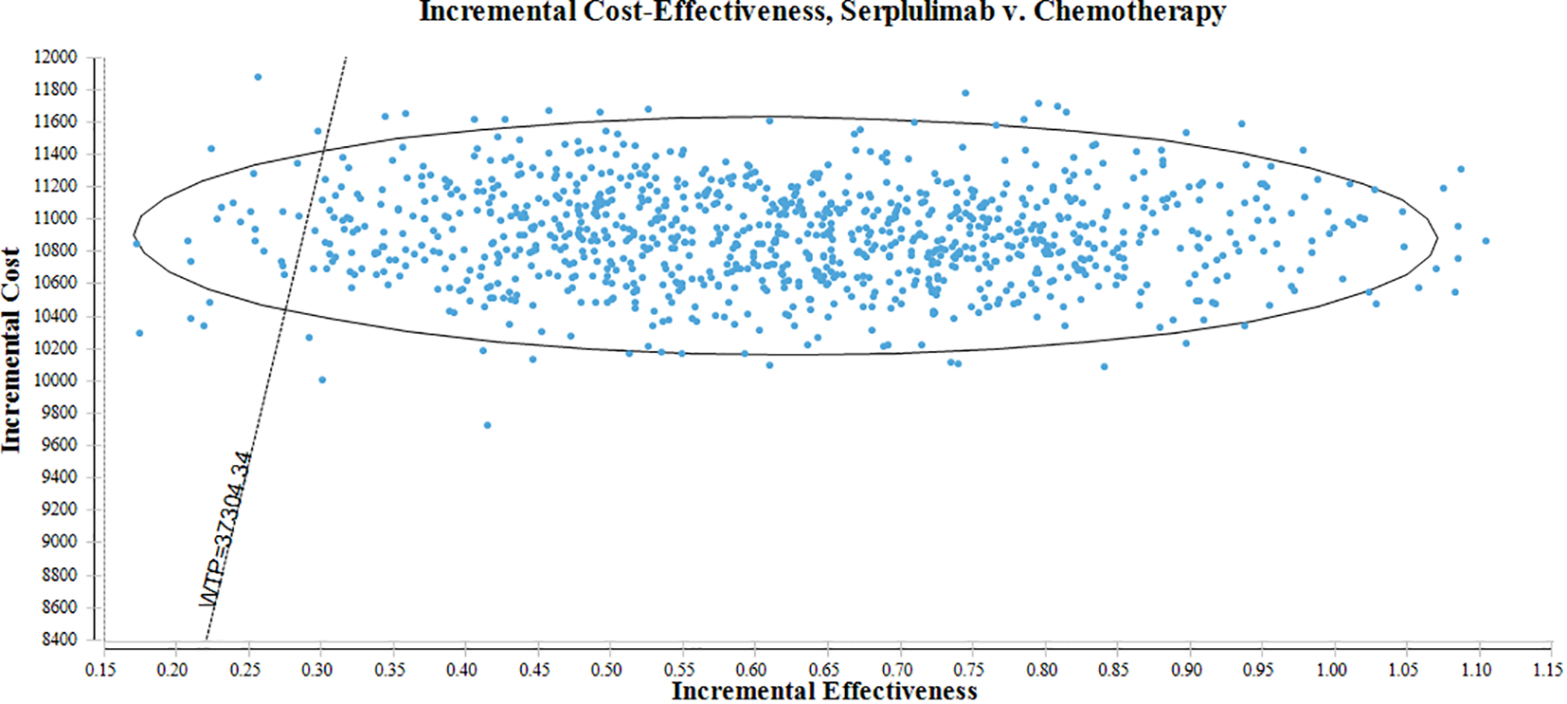
Scatterplot of the Monte Carlo simulation.

**Figure 3** displays a scatterplot depicting the outcomes of the Monte Carlo simulation. It is important to emphasize that the diagonal line in the figure represents the WTP value, which is the key threshold for determining whether the benefits of serplulimab outweigh the associated costs. Significantly, at the WTP threshold of $37,304.346 per QALY, there is a notably high probability, approximately 97.40%, that the group that receives serplulimab is considered the more cost-effective treatment option.

## 4 Discussion

Lung cancer has emerged as the leading cause of cancer-related mortality worldwide, and it is estimated to cause approximately 1.8 million deaths in 2020, accounting for approximately 18% of all cancer fatalities (23). In addition, it is noteworthy to highlight that a significant proportion of recently identified instances of lung cancer, amounting to more than one-third of all reported cases, along with a staggering 40% of corresponding fatalities, are recorded within the borders of China (24). This disconcerting trend is expected to escalate in the foreseeable future, thereby posing a significant challenge to public health initiatives in China (25). Moreover, health economics studies have elucidated the intricate nature of the economic impact attributable to lung cancer. It is crucial to recognize that the economic burden of this devastating disease extends beyond the direct costs associated with medical treatment and care. The survey conducted by Liu et al. in 2021 revealed that lung cancer patients and their families bear a significant economic burden from a variety of sources (26). This prevailing economic burden not only detrimentally impacts the quality of life of those directly affected but also hampers the overall socio-economic progress and development of the nation.

Recently, the ASTRUM-005 clinical trial has revealed significant findings regarding the efficacy of serplulimab in prolonging PFS and OS of patients with ES-SCLC. These positive results, coupled with the absence of any new safety concerns, shed light on the potential of serplulimab as a promising first-line treatment option for ES-SCLC. However, the high cost associated with serplulimab may pose a substantial barrier to widespread adoption, particularly for economically disadvantaged patients. In China, where socioeconomic disparities often hinder equal access to both outpatient and inpatient care for cancer patients, it becomes imperative to prioritize value-based oncological care (27). Hence, it becomes imperative to undertake comprehensive economic evaluations within the realm of drug treatment options, with the aim of comprehending the intricate dynamics of costs and benefits accompanying these options, thereby facilitating the identification of efficacious and economical treatment. Therefore, the primary aim of this study is to evaluate the cost-effectiveness of incorporating serplulimab as a first-line treatment strategy for ES-SCLC from the perspective of the Chinese healthcare system.

Based on the findings of our current study, the combined administration of serplulimab and chemotherapy demonstrated a favorable outcome, resulting in a total of 1.172 QALYs at a cost of $31020.152. In comparison to the chemotherapy-only arm, the group receiving serplulimab showed an incremental gain of 0.626 QALYs with incremental costs amounting to $10893.995. This calculated to an ICER of $17402.548 per QALY gained. Importantly, the ICER in our study falls below the commonly accepted WTP threshold of $37304.346 per QALY. This suggests that the utilization of serplulimab in the treatment of the condition under investigation could be considered a cost-effective therapeutic option in China.

Our finding contributes to the body of evidence surrounding the cost-effectiveness of serplulimab in combination with chemotherapy. By providing robust evidence to support its potential value, our study encourages further exploration and consideration of serplulimab’s role in the management of this condition. The cost-effectiveness analysis underscores the potential benefits of adopting serplulimab, thereby informing health policy decisions and empowering healthcare practitioners to make informed clinical choices in the best interest of their patients.

The findings from the sensitivity analysis underscore the significant influence of several factors on the outcomes, including the cost of best supportive care, the utility of PFS, the subsequent costs, the utility of PD, the cost of serplulimab, and the PFS utility. However, it is imperative to highlight that even when these parameters were varied by ±25%, they did not substantially alter the overall findings of the analysis. This observation holds substantial importance as it indicates the robustness of the model and suggests that the results are not highly susceptible to changes in these aforementioned parameters.

Previous studies have extensively examined the pharmacoeconomics of ICIs in combination with chemotherapy as a first-line treatment for ES-SCLC. One notable study conducted by Zhu et al. compared the cost-effectiveness of pembrolizumab, as a first-line treatment for ES-SCLC patients from the perspective of US payers. Their findings indicated that pembrolizumab may not be a financially viable option (28). Furthermore, Ionova et al. developed a Markov model to evaluate the cost-effectiveness of two other ICIs, durvalumab and atezolizumab. Their analysis revealed that atezolizumab, in comparison to durvalumab, provided greater health and cost benefits for ES-SCLC patients in the United States (29). Moreover, an important study by Wang et al. investigated the cost-effectiveness of adebrelimab, a different therapeutic approach, in comparison to chemotherapy for ES-SCLC patients. Their results suggested that adebrelimab in combination with chemotherapy might be a cost-effective strategy for the first-line treatment of ES-SCLC from the Chinese healthcare system (30). The aforementioned studies have made significant contributions to the existing body of research on the cost-effectiveness analysis of diverse therapeutic approaches, thus offering valuable insights into the economic viability of ICIs as a treatment option for ES-SCLC. The economic implications of utilizing ICIs in combination with chemotherapy for ES-SCLC represent a crucial area of study, and the findings from these scholarly investigations shed light on the cost-effectiveness and feasibility of such treatment approaches (31). Further research is warranted to explore additional factors influencing the pharmacoeconomics of ES-SCLC treatments, taking into account various healthcare systems and cost thresholds.

There are several limitations that need to be acknowledged in this study. Firstly, the lack of head-to-head data prevented us from including other ICIs such as atezolizumab and durvalumab, which have demonstrated notable improvements in PFS and OS. This omission may limit the comprehensiveness of our findings and highlights the need for further research in this area. Secondly, it is important to recognize the potential biases associated with the original clinical trial upon which our study is based. The absence of long-term follow-up data for patients enrolled in the ASTRUM-005 trial introduces a level of uncertainty and may influence the predicted outcomes derived from our simulations. To mitigate the impact of this limitation, we set the simulation period to 10 years. However, it is important to acknowledge that this might not fully account for the long-term effects and long-lasting benefits of the treatment. Furthermore, the exclusion of immune-related adverse events (AEs) and grade 1 or 2 AEs in our analysis could lead to an overestimation of the results associated with serplulimab. Future studies should consider incorporating these AEs to provide a more comprehensive evaluation of the treatment’s efficacy and safety profile. Nevertheless, the results of our sensitivity analyses suggest that the limited impact of these AEs on our findings does not undermine our overall conclusions. Additionally, it is worth noting that our study assumed certain patient characteristics such as a weight of 60 kg, creatinine clearance of 70 μmol/L, and a body surface area of 1.72 m², which might introduce potential biases when applied to real-world clinical practice. To address this concern, we conducted robustness analyses by varying these assumptions within a range of ±25%, ensuring the robustness and generalizability of our results. Lastly, it is important to acknowledge that our analysis focused solely on the direct medical care costs associated with serplulimab treatment, neglecting indirect costs such as loss of productivity and caregiver expenses. Future studies should aim to include these indirect costs to provide a more comprehensive economic evaluation of the treatment.

## 5 Conclusion

In China, the utilization of serplulimab in combination with chemotherapy for the treatment of ES-SCLC has demonstrated superior cost-effectiveness when compared to chemotherapy alone, making it may be a key frontline treatment option in clinical practice.

## Data availability statement

The original contributions presented in the study are included in the article/**Supplementary Material**. Further inquiries can be directed to the corresponding author.

## Author contributions

ZZ: Writing – original draft, Writing – review & editing, Resources. HCC: Data curation, Software, Investigation, Writing – review & editing. HFC: Project administration, Supervision, Validation, Visualization, Writing – review & editing.

## Supplementary material

Supplementary Figure 1
**(A)** Modes simulation visual overall survival curve of serplulimab group; **(B)** Modes simulation visual overall survival curve of chemotherapy group; **(C)** Modes simulation visual progression-free survival curve of serplulimab group; **(D)** Modes simulation visual progression-free survival curve of chemotherapy group.
