# Supplementary material for: Cost-effectiveness analysis of serplulimab combination therapy versus chemotherapy alone for patients with extensive-stage small cell lung cancer
Source: Front Oncol. 2024 Jan 11;13:1259574. doi: 10.3389/fonc.2023.1259574 (PMC10812113; doi:10.3389/fonc.2023.1259574)
Supplement: Supplementary file 1 [file Table_1.docx]

**Supplementary Table 1. Comparison of survival models**

|  | AIC | | BIC | |
| --- | --- | --- | --- | --- |
|  | Serplulimab group | Chemotherapy group | Serplulimab group | Chemotherapy |
| PFS |  |  |  |  |
| Weibull | 1134.014 | 610.042 | 1141.942 | 616.598 |
| **Log-logistic** | **1115.596** | **597.242** | **1123.523** | **603.799** |
| Log-normal | 1113.798 | 605.280 | 1121.725 | 611.836 |
| Gompertz | 1164.101 | 633.935 | 1172.028 | 640.491 |
| Exponential | 1180.558 | 652.323 | 1184.521 | 655.601 |
| Gamma | 1124.444 | 604.266 | 1132.371 | 610.822 |
| OS |  |  |  |  |
| Weibull | 1009.651 | 607.775 | 1017.578 | 614.331 |
| **Log-logistic** | **1007.935** | **604.823** | **1015.863** | **611.380** |
| Log-normal | 1013.414 | 605.738 | 1021.341 | 612.294 |
| Gompertz | 1020.336 | 616.734 | 1028.263 | 623.290 |
| Exponential | 1043.123 | 625.446 | 1047.086 | 628.724 |
| Gamma | 1008.905 | 606.044 | 1016.832 | 612.600 |

AIC: Akaike information criterion; BIC: Bayesian Information Criterion; OS: Overall survival; PFS: Progression-free survival;
