# Supplementary figures and images for: Cost-effectiveness analysis of serplulimab combination therapy versus chemotherapy alone for patients with extensive-stage small cell lung cancer
Source: Front Oncol. 2024 Jan 11;13:1259574. doi: 10.3389/fonc.2023.1259574 (PMC10812113; doi:10.3389/fonc.2023.1259574)

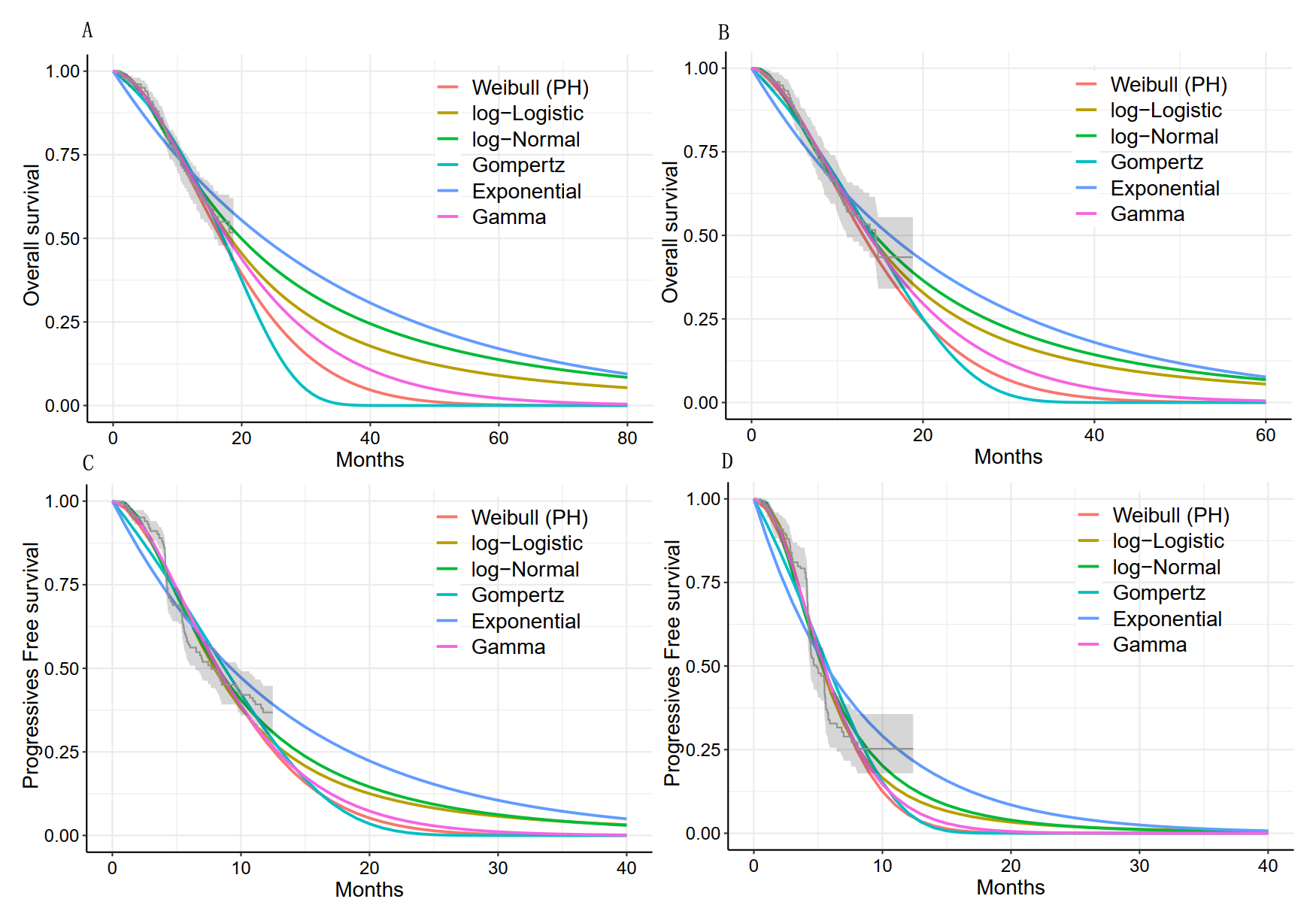

Supplement: Supplementary Figure 1 — (A) Modes simulation visual overall survival curve of serplulimab group; (B) Modes simulation visual overall survival curve of chemotherapy group; (C) Modes simulation visual progression-free survival curve of serplulimab group; (D) Modes simulation visual progression-free survival curve of chemotherapy group. [file Image_1.tif]
